# Clinical Predictors of Heart Failure and Short-Term Outcomes in Frail Adults: A Single-Center Retrospective Cohort Study

**DOI:** 10.7759/cureus.115053

**Published:** 2026-08-23

**Authors:** Eranda L Ranasinghe Arachchi, Pavanthi Ranasinghe Arachchi, Haider Khan, Yee Mon Wint

**Affiliations:** 1 Department of Medicine, Health Education England, Cambridge, GBR; 2 Department of Science, Anglia Ruskin University, Cambridge, GBR; 3 School of Medicine, Northern Ireland Medical and Dental Training Agency, Belfast, GBR; 4 Department of Geriatric Medicine, East Suffolk and North Essex NHS Foundation Trust, Ipswich, GBR

**Keywords:** clinical frailty, geriatric medicine, heart failure in elderly, nt-probnp, prognostic indicators

## Abstract

Introduction

Heart failure (HF) remains a leading cause of morbidity and mortality in frail adults. Traditional prognostication relies on ejection fraction and cardiac biomarkers, yet this approach may not adequately capture multifactorial vulnerability in frail populations. Although N-terminal pro-B-type natriuretic peptide (NT-proBNP) along with ejection fraction phenotype has emerged as an important risk stratification marker, its prognostic utility in specific populations remains incompletely characterized. Frailty is increasingly recognized as a critical determinant of prognosis in HF patients. The interrelationship between biomarker elevation, frailty, and short-term outcomes in hospitalized older adults requires investigation.

Methods

This retrospective observational cohort study was conducted at a secondary care hospital in the United Kingdom between September and December 2024, utilizing data from 240 true HF admissions from a local audit and service improvement initiative. The study enrolled 98 consecutive eligible admissions with confirmed Rockwood Clinical Frailty Score ≥ 5, acute HF diagnosis, and complete 12-week follow up data. Frailty was measured using the Rockwood Clinical Frailty Scale, and NT-proBNP was measured using standard laboratory assays. Ejection fraction parameters were obtained from admission echocardiographic assessment. Pre-existing cardiovascular risk factors-atrial fibrillation (AF), chronic kidney disease (CKD), and coronary artery disease (CAD)-were gathered retrospectively on a case-by-case basis. The composite primary outcome was death or hospitalization for acute HF within 12 weeks. Statistical analyses included Mann-Whitney U tests, chi-squared analyses, binary logistic regression, and receiver operating characteristic (ROC) analysis.

Results

The cohort comprised 98 adults (mean age 76.4 years; Frailty Scale 5.38) with a mean admission NT-proBNP of 5,060 pg/mL. CKD, AF, and CAD were present in 20.4%, 32.7%, and 33.7%, respectively. HF phenotypes included 41.5% in HF with preserved ejection fraction (HFpEF), 35.4% in HF with mildly reduced ejection fraction (HFmrEF), and 23.1% in HF with reduced ejection fraction (HFrEF). Adverse outcomes occurred in 33.7%. Age was significantly associated with poor prognosis. Despite limited multivariable analysis due to small sample size, NT-proBNP and frailty showed directional associations. Multivariable logistic regression identified no independent predictors after adjustment. ROC analysis showed acceptable discrimination by admission NT-proBNP (area under the curve (AUC) 0.703).

Conclusion

Short-term prognosis in frail HF populations reflects complex multifactorial vulnerability rather than isolated biomarker elevation. Although NT-proBNP demonstrated acceptable discriminatory ability, the absence of independent multivariable predictors indicates that prognostication cannot rely on isolated cardiac biomarkers alone. Rather, outcomes are fundamentally determined by age-related physiological decline, comorbidity burden, neurohormonal activation, and reduced physiological reserve. Furthermore, ejection fraction phenotype was not significantly associated with adverse outcome and showed that the vulnerability to acute stressors often eclipses isolated ventricular metrics. These findings emphasize incorporating comprehensive assessment principles into HF prognostication rather than adhering to HF guidelines.

## Introduction

Heart failure remains a leading cause of morbidity and mortality in older adults, with acute decompensation requiring hospitalization accounting for substantial healthcare burden and resource utilization [1]. Among hospitalized populations, older adults often exhibit complex clinical presentations complicated by multiple chronic conditions, reduced physiological reserve, and increased vulnerability to adverse outcomes [2]. Despite advances in heart failure therapies, short-term prognosis following acute hospitalization remains difficult to predict, particularly in heterogeneous older populations [3].

Traditional prognostication in heart failure has relied heavily on ejection fraction (EF) phenotype and cardiac-specific biomarkers; however, this approach may not adequately capture the complex multifactorial vulnerability that drives outcomes in frail older populations [4]. Natriuretic peptides, specifically N-terminal pro-B-type natriuretic peptide (NT-proBNP), have emerged as important biomarkers for heart failure diagnosis, risk stratification, and prognosis [5]. Elevated NT-proBNP reflects heightened neurohormonal activation and correlates with hemodynamic derangement, ventricular wall stress, and disease severity. However, the prognostic utility of NT-proBNP in specific populations remains incompletely characterized. Frailty, a syndrome characterized by diminished physiological reserve, increased vulnerability to stressors, and heightened susceptibility to adverse health outcomes, has increasingly been recognized as an important determinant of prognosis in older heart failure patients [6]. Yet, the interrelationship between biomarker elevation, frailty, and short-term outcomes in older hospitalized heart failure populations remains poorly understood.

The relative contributions of age, frailty burden, renal function, comorbidities, and neurohormonal activation to short-term mortality and readmission risk in this population warrant investigation. Therefore, this study primarily examined whether admission NT-proBNP independently predicts short-term adverse outcomes in frail adults hospitalized with acute heart failure. The primary hypothesis was that "admission NT-proBNP independently predicts short-term adverse outcomes in frail adults hospitalized with acute heart failure." While evaluating the primary hypothesis, the effects of additional parameters, such as EF phenotype, frailty, and cardiovascular risk factors, on acute heart failure mortality and morbidity were also evaluated as secondary factors.

## Materials and methods

Study design

This was a retrospective observational cohort study conducted at a single secondary care hospital in the United Kingdom. The study used anonymized data originally collected as part of a local clinical audit and service improvement project evaluating outcomes in older adults admitted with acute heart failure. Following completion of the audit, the dataset was retrospectively analyzed to examine the association between heart failure phenotype and short-term clinical outcomes. Appropriate institutional approvals for secondary use of anonymized audit data were obtained in accordance with local clinical governance procedures at the Research Development Committee-South Eastern Health and Social Care Trust, Northern Ireland.

Study population and inclusion and exclusion criteria

This retrospective observational cohort study was conducted at a secondary care hospital in the United Kingdom between September and December 2024, utilizing data from 564 admissions with suspicious heart failure from a local clinical audit and service improvement initiative. These 564 admissions were screened and filtered down to 240 true acute heart failure admissions, which were evaluated and analyzed in this study.

After removing the admissions that were merely mimicking heart failure (exacerbation of asthma or chronic obstructive pulmonary disease, lymphedema, acute coronary syndrome (ACS), and pure fluid overload due to missed dialysis sessions), a total of 240 patients were identified and evaluated for eligibility. Patients were included if they met all of the inclusion criteria. Inclusion criteria included age more than 35 years, confirmed diagnosis of acute heart failure, Rockwood Clinical Frailty Score of 5 or above, and complete follow-up data for all-cause mortality and heart failure readmission within 12 weeks of index admission.

Patients were excluded if they did not meet the predefined inclusion criteria or if key clinical data were unavailable. Specifically, exclusions included patients without a confirmed diagnosis of acute heart failure, age ≤ 35 years, or Rockwood Clinical Frailty Score of less than 5. Patients with missing 12-week follow-up data for mortality or heart failure readmission were excluded from outcome analyses. To avoid duplication, only the index admission during the study period was included, and subsequent readmissions were excluded.

**Figure 1:**
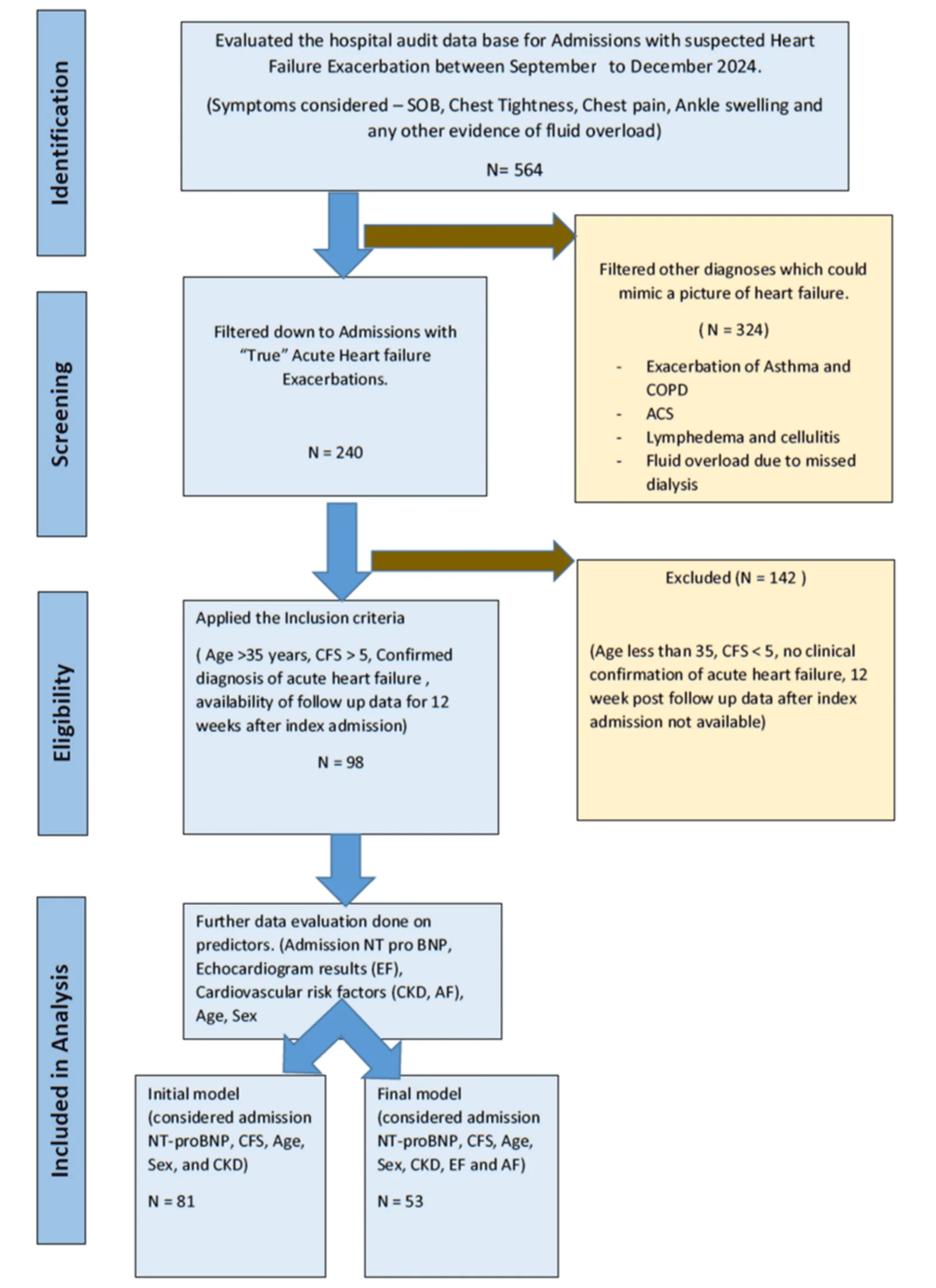
Participant flow diagram The participant flow diagram represents participant recruitment and the filtering process through the entire study. The flow of patients from initial hospital audit through screening, eligibility assessment, and inclusion to the final phase is indicated. SOB: shortness of breath; COPD: chronic obstructive pulmonary disease; ACS: acute coronary syndrome; CFS: Clinical Frailty Scale; NT-proBNP: N-terminal pro-B-type natriuretic peptide; CKD: chronic kidney disease; EF: ejection fraction; AF: atrial fibrillation

A total of 98 consecutive eligible admissions met the predefined inclusion criteria and were included in the final cohort. Admission NT-proBNP levels, echocardiographic assessment of left ventricular EF, and frailty scores were recorded as study variables; however, these parameters were not mandatory for cohort inclusion, and data availability varied within the study population. NT-proBNP measurements were available in 86 patients and echocardiographic data in 65 patients. Missing data reflected routine clinical practice and the retrospective nature of the study, arising from investigations not being performed in all patients, unavailable echocardiographic assessments, or incomplete documentation within the electronic medical record. Figure 1 presents the participant flow diagram illustrating the screening and filtering process for the study's clinical cases.

Data collection and definitions

Clinical and demographic data were extracted from electronic medical records and the hospital heart failure audit database. Variables collected included age, sex, and cardiovascular comorbidities such as chronic kidney disease (CKD), atrial fibrillation (AF), and coronary artery disease/ischemic heart disease (CAD/IHD).

Heart failure phenotype was classified according to transthoracic echocardiography performed within 72 hours of index admission or from a recent clinically valid assessment. Patients were categorized into heart failure with preserved EF (HFpEF), where EF is above 50%; heart failure with mildly reduced EF (HFmrEF), where EF is 41%-49%; and heart failure with reduced EF (HFrEF), where EF is recorded as 40% or less.

Admission NT-proBNP concentration was measured from blood samples obtained upon hospital admission using standard laboratory assays. Raw NT-proBNP values were recorded in pg/mL. Due to marked positive skewness and non-normal distribution of raw NT-proBNP values, logarithmic transformation was performed prior to inferential statistical analyses to improve distributional symmetry and reduce the influence of extreme observations.

Frailty was measured using the Rockwood Clinical Frailty Scale (CFS) by Rockwood et al. [7], a validated nine-point assessment tool and the global standard for assessing individuals regarding comorbidity, disability, and dependence. The CFS scores were extracted from routine clinical documentation or assigned at admission by clinicians according to the institution's standard frailty assessment practices. As this was a retrospective study using routinely collected clinical data, formal face-to-face assessment and inter-rater variability were not performed.

The composite primary outcome was defined as death or hospitalization for acute heart failure exacerbation requiring readmission within 12 weeks of the initial admission date. Outcome status was determined from medical records, hospital administrative databases, and follow-up assessments.

Statistical analysis

Statistical analyses were performed using Jamovi 2.6.44 software. Baseline demographic, clinical, and laboratory characteristics of the total sample were summarized using means and standard deviations (SDs) for continuous variables and frequencies with percentages for categorical variables. Normality of continuous variables was assessed using the Shapiro-Wilk test. Visual inspection of histograms and Q-Q plots was additionally performed to assess distributional characteristics and identify potential skewness or extreme observations. Given non-normal distributions, group comparisons were conducted using non-parametric tests. Homogeneity of variance was evaluated using Levene's test.

Mann-Whitney U tests were performed to compare continuous variables between participants who experienced the composite outcome and those who did not. Chi-squared analyses were used to examine associations between categorical clinical variables (sex, CKD, AF, CAD/IHD, and EF phenotype) and outcome status.

Multicollinearity among the predictor variables was assessed using variance inflation factors (VIFs). VIF values were 1.09 for log-transformed NT-proBNP and 1.04 for AF, CKD, and EF phenotype, with corresponding tolerance values ranging from 0.916 to 0.961. As all VIF values were substantially below the conventional threshold of 5, there was no evidence of problematic multicollinearity among the predictors.

Binary logistic regression was performed to evaluate whether admission NT-proBNP independently predicted the composite outcome after adjustment for age, frailty score, sex, CKD, AF, and EF phenotype. Adjusted odds ratios (ORs) with 95% confidence intervals (CIs) and associated p-values were computed for each predictor. Model fit was assessed using McFadden's R².

Receiver operating characteristic (ROC) analysis was conducted to evaluate the discriminatory ability of admission log-transformed NT-proBNP for predicting the composite outcome. The area under the curve (AUC) was calculated with interpretation based on standard conventions. Statistical significance was set at α = 0.05 for all tests. Adjustment for multiple comparisons was not performed, as the univariate analyses were considered exploratory and intended to identify candidate variables for multivariable modeling rather than to test prespecified independent hypotheses.

Predictors for the multivariable logistic regression model were selected a priori, based on their clinical relevance to short-term adverse outcomes and the primary objectives of the study, rather than solely on univariate statistical significance. Given the relatively small complete-case sample, the multivariable analysis was considered exploratory, and the estimates may be susceptible to limited statistical power. This is a major limitation we have identified, which is further elaborated in the Discussion section.

## Results

Participant characteristics

A total of 98 older adults admitted with acute heart failure were included in the analysis. The mean age of the cohort was 76.4 years (SD = 12.6), with participant ages ranging from 39 to 97 years. Men represented 54.1% of the sample (n = 53), while women accounted for 45.9% (n = 45). The mean CFS score was 5.38 (SD = 1.23), indicating a moderately frail inpatient population.

Admission NT-proBNP was available for 86 patients and had a median of 2,793 pg/mL (IQR of 1,045-6,325 pg/mL with a mean of 5,060 pg/mL). Due to marked positive skewness of NT-proBNP values, logarithmic transformation was applied for inferential analyses. The mean log-transformed NT-proBNP value was 7.66 (SD = 1.54).

CKD was present in 20.4% of participants (n = 20), AF in 32.7% (n = 32), and CAD/IHD in 33.7% (n = 33). Echocardiographic data were available for 65 patients, comprising 27 (41.5%) with HFpEF, 23 (35.4%) with HFmrEF, and 15 (23.1%) with HFrEF.

Normality testing

Normality assumptions for continuous variables were assessed using the Shapiro-Wilk test. Age (W = 0.965, p = 0.010), log-transformed NT-proBNP (W = 0.963, p = 0.015), and frailty score (W = 0.956, p = 0.003) significantly deviated from normality. Consequently, non-parametric Mann-Whitney U tests were used for comparisons between outcome groups.

Univariate analysis

Patients who experienced the composite outcome of death or heart failure readmission were significantly older than those without adverse outcomes. The mean age of patients with an adverse outcome was 80.8 years (SD = 10.1), compared with 74.2 years (SD = 13.2) among those without adverse events. Mann-Whitney U analysis demonstrated a significant association between older age and adverse outcome, U = 737, p = 0.012.

Mean log-transformed NT-proBNP levels were higher in the outcome group (M = 8.01, SD = 1.40) compared with the non-outcome group (M = 7.48, SD = 1.60), although this difference did not reach statistical significance (U = 658, p = 0.100). Similarly, patients with adverse outcomes demonstrated higher frailty scores (M = 5.64, SD = 1.19) compared with patients without adverse outcomes (M = 5.23, SD = 1.24), with a trend toward significance (U = 784, p = 0.087). The study noted that patients experiencing adverse outcomes exhibited numerically higher admission log-transformed NT-proBNP values and greater frailty scores than those without adverse outcomes.

Categorical associations

Chi-squared analyses were performed to examine associations between categorical clinical variables and the composite outcome. No significant association was observed between sex and adverse outcome, χ²(1) = 0.13, p = 0.716. Similarly, CKD was not significantly associated with death or readmission within 12 weeks, χ²(1) = 0.45, p = 0.502. CAD/IHD also demonstrated no significant relationship with outcome status, χ²(1) = 0.25, p = 0.615.

Patients with AF (n = 32) demonstrated a higher proportion of adverse outcomes (14/32) compared with patients without AF (n = 66) who had adverse outcomes (18/66), 43.8% vs. 27.3%, χ²(1) = 2.16, p = 0.142. Adverse outcomes occurred in six of 23 patients (26.1%) with HFmrEF, seven of 27 (25.9%) with HFpEF, and three of 15 (20.0%) with HFrEF. Outcome frequencies did not differ significantly between EF phenotypes, χ²(2) = 0.38, p = 0.828.

Multivariable logistic regression

Binary logistic regression was performed to evaluate whether admission NT-proBNP independently predicted the composite outcome after adjustment for relevant clinical variables including age, frailty, sex, CKD, AF, and EF phenotype. The overall regression model demonstrated modest explanatory power (McFadden R² = 0.143).

Age was positively associated with adverse outcomes after adjustment, although this association did not reach statistical significance (OR = 1.04, 95% CI (0.96, 1.12), p = 0.370). Similarly, higher log-transformed NT-proBNP levels were directionally associated with increased odds of adverse outcomes (OR = 1.15, 95% CI (0.68, 1.94), p = 0.608). Frailty scores also demonstrated a positive directional association with outcome (OR = 1.38, 95% CI (0.74, 2.58), p = 0.315).

The overall likelihood-ratio test for the multivariable logistic regression model was non-significant, χ²(7) = 5.72, p = 0.573, indicating that the full set of predictors did not significantly improve model fit compared with the intercept-only model. Individual likelihood-ratio tests were also non-significant for age (p = 0.355), log-transformed NT-proBNP (p = 0.606), frailty/CFS (p = 0.298), CKD (p = 0.292), AF (p = 0.693), and EF group (p = 0.927).

The Hosmer-Lemeshow goodness-of-fit test was also non-significant, χ²(8) = 7.19, p = 0.516. This indicates that there was no statistically significant evidence of poor fit between the model-predicted probabilities and the observed outcomes, suggesting acceptable calibration. However, we noted that this finding should be interpreted cautiously because the final complete-case model included only 53 participants, which is a major limitation of this study.

**Figure 2:**
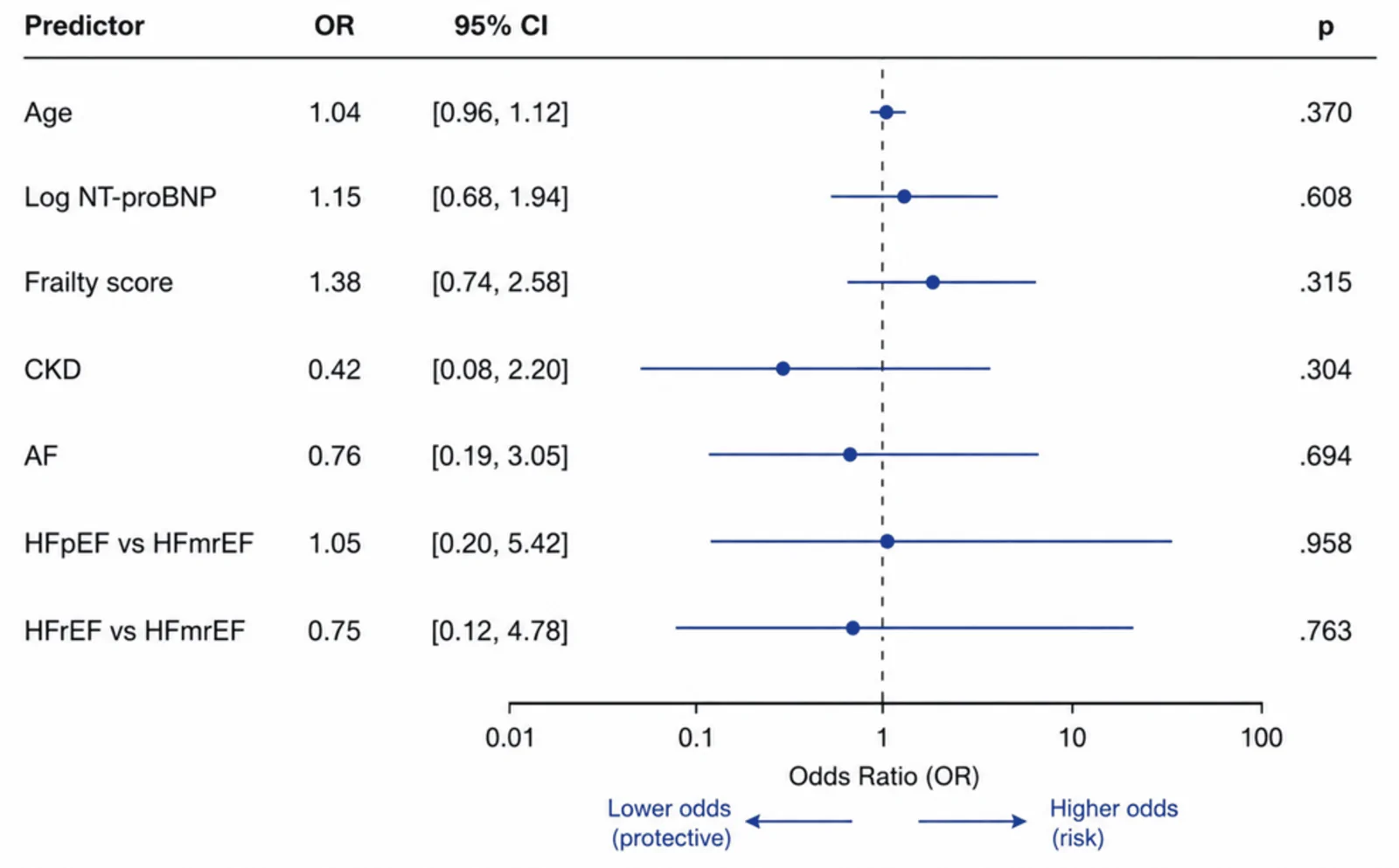
Categorical predictors and composite outcome associations Forest plot of adjusted odds ratios (ORs) from multivariable logistic regression. Adjusted ORs and 95% confidence intervals (CIs) are shown for demographic, clinical, biochemical, and heart failure phenotype variables. No independent predictors of the outcome were identified following multivariable adjustment. Although frailty demonstrated the strongest positive association with the outcome, all CIs crossed the null value (OR = 1), indicating statistical non-significance. Wide CIs suggest limited precision and possible underpowering of the analysis. NT-proBNP: N-terminal pro-B-type natriuretic peptide; CKD: chronic kidney disease; AF: atrial fibrillation; HFpEF: heart failure with preserved ejection fraction; HFmrEF: heart failure with mildly reduced ejection fraction; HFrEF: heart failure with reduced ejection fraction

Figure 2 shows categorical predictors and composite associations of the variables that were considered.

**Figure 3:**
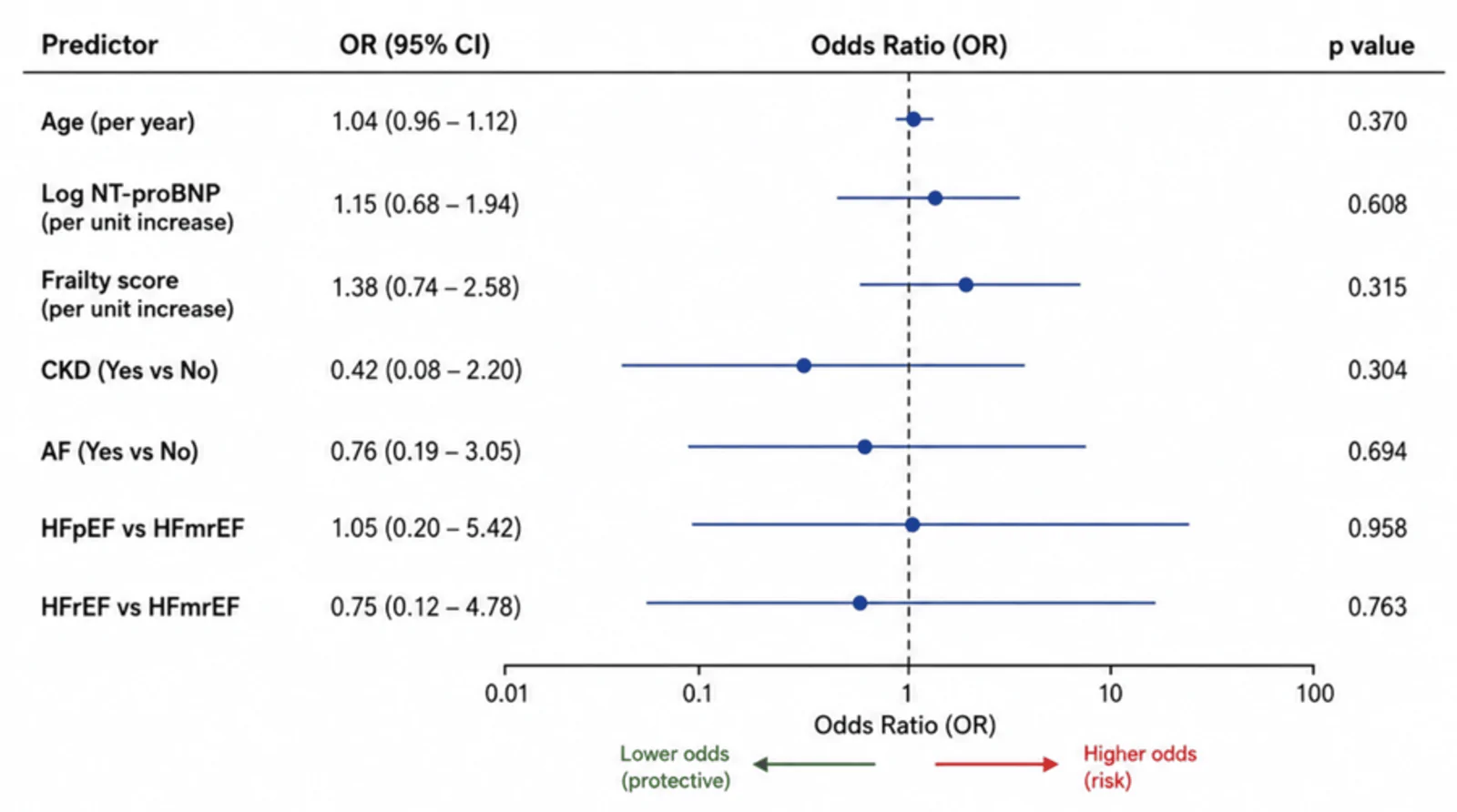
Summary of multivariable logistic regression. Association of clinical and biomarker variables with outcome In the multivariable logistic regression analysis, no clinical, biochemical, or heart failure phenotype variable demonstrated an independent association with the outcome. Although frailty exhibited the strongest positive effect estimate (OR 1.38), its confidence interval (CI) crossed unity, precluding statistical significance. Similarly, age and NT-proBNP showed modest positive associations, whereas CKD and AF demonstrated point estimates below unity; however, all estimates were accompanied by wide CIs. NT-proBNP: N-terminal pro-B-type natriuretic peptide; CKD: chronic kidney disease; AF: atrial fibrillation; HFpEF: heart failure with preserved ejection fraction; HFmrEF: heart failure with mildly reduced ejection fraction; HFrEF: heart failure with reduced ejection fraction; OR: odds ratio

Figure 3 shows the summary of multivariable logistic regression between categorical predictors and the outcome.

ROC analysis

**Figure 4:**
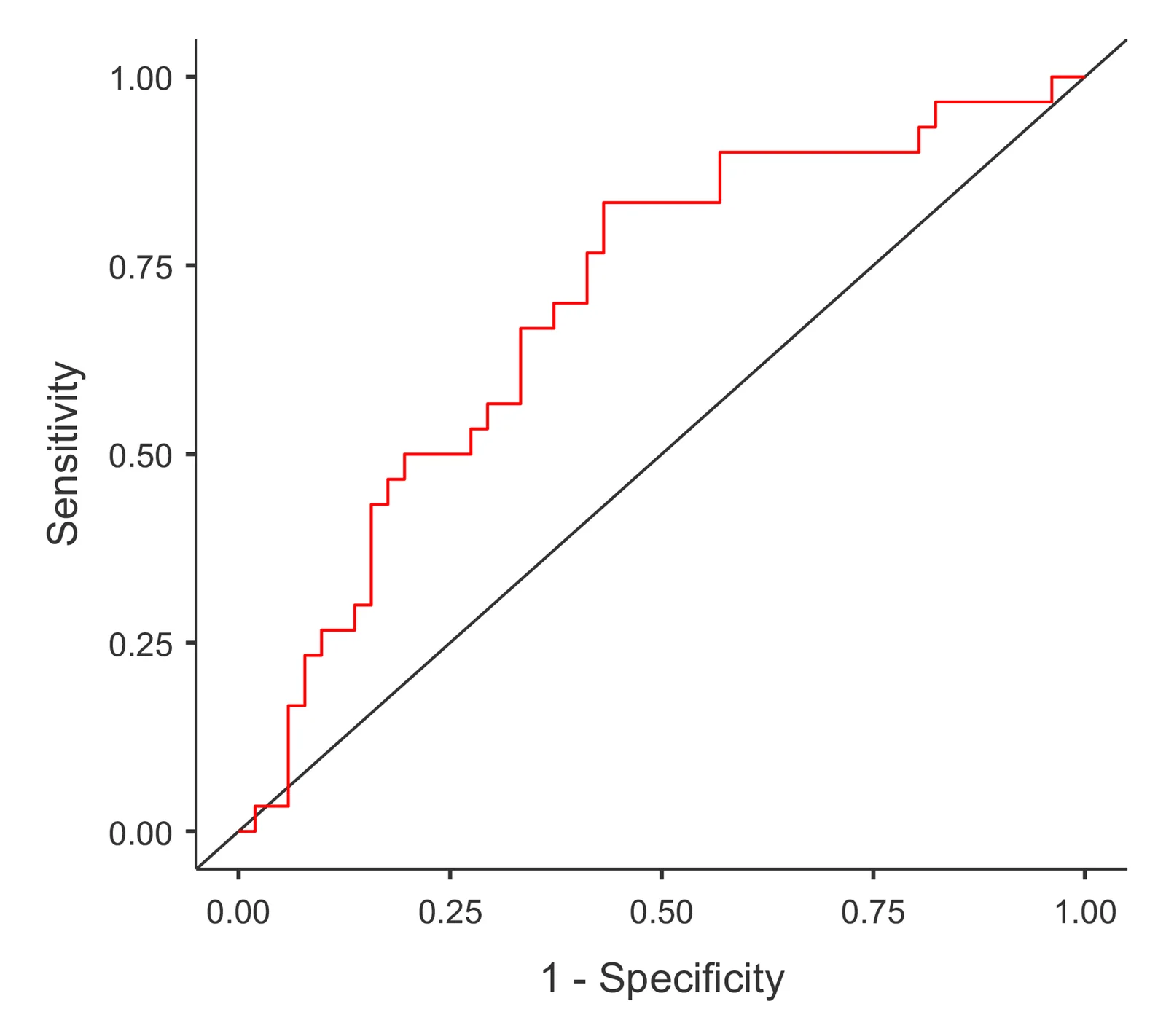
The initial model, including age, sex, chronic kidney disease, Rockwood Clinical Frailty Score, and NT-proBNP Receiver operating characteristic (ROC) curve for the initial multivariable logistic regression model predicting the composite outcome of death or heart failure readmission within 12 weeks using age, sex, frailty score, chronic kidney disease, and admission log-transformed NT-proBNP. The ROC curve remained above the diagonal reference line, demonstrating discriminatory ability greater than chance, with an area under the curve (AUC) of 0.700, indicating acceptable prognostic performance (95% confidence interval (0.544, 0.861), p = 0.002). NT-proBNP: N-terminal pro-B-type natriuretic peptide

ROC analysis was conducted to evaluate the discriminatory performance of admission log-transformed NT-proBNP for predicting the composite outcome. Multivariable logistic regression analyses were performed using complete-case analysis. The initial model, including age, sex, CKD, Rockwood Clinical Frailty Score, and NT-proBNP, included 81 patients with complete data, as indicated in Figure 4.

The multivariable logistic regression included 81 complete cases, of whom 30 (37.0%) experienced the composite outcome and 51 (63.0%) did not. With five predictors included in the model, this corresponded to approximately six outcome events per predictor variable.

**Figure 5:**
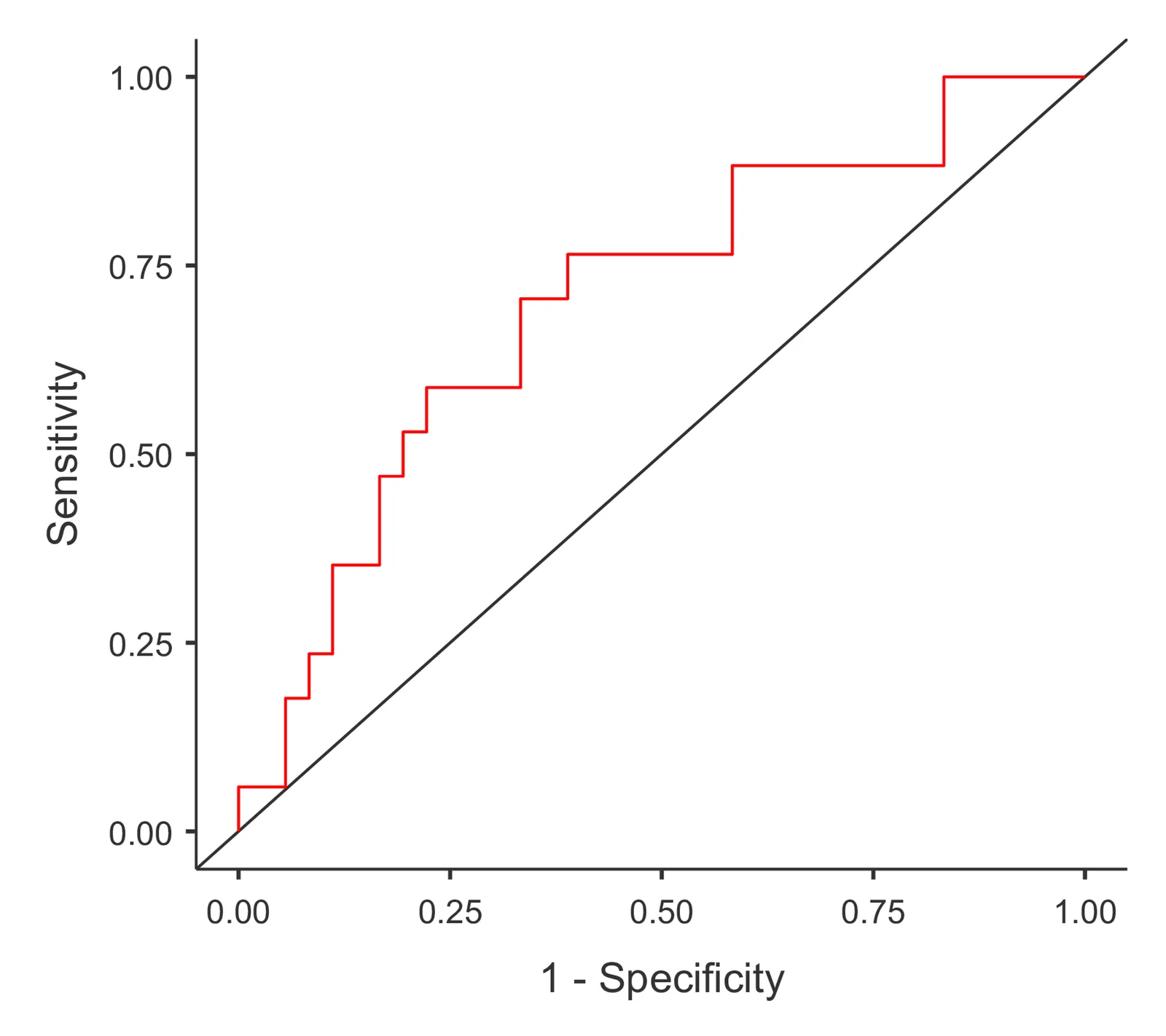
Fully adjusted model incorporating atrial fibrillation and ejection fraction Receiver operating characteristic (ROC) curve for the expanded multivariable logistic regression model predicting the composite outcome of death or heart failure readmission within 12 weeks using age, frailty score, chronic kidney disease, atrial fibrillation, ejection fraction phenotype, and admission log-transformed NT-proBNP. The ROC curve remained above the diagonal reference line, demonstrating acceptable discriminatory performance with an area under the curve (AUC) of 0.703. NT-proBNP: N-terminal pro-B-type natriuretic peptide

Figure 5 shows a fully adjusted model incorporating AF and EF, which was restricted to 53 patients with complete covariate data. ROC analysis demonstrated acceptable discriminatory ability, with an AUC of 0.703 (95% CI (0.578, 0.822), p = 0.001).

## Discussion

The present study investigated the association between admission NT-proBNP levels and short-term adverse outcomes in frail older adults hospitalized with acute heart failure. The primary aim was to determine whether admission NT-proBNP independently predicted the composite outcome of death or heart failure readmission within 12 weeks while accounting for important clinical variables including EF phenotype, age, frailty, renal dysfunction, and AF.

Admission NT-proBNP and frailty demonstrated clinically meaningful directional associations with adverse outcome, although these associations did not reach statistical significance following multivariable adjustment. The descriptive plots further supported the interpretation that this was a clinically heterogeneous frail heart failure cohort. Raw NT-proBNP values demonstrated marked positive skewness with several extreme high-value observations, suggesting variation in hemodynamic and neurohormonal burden across patients. Logarithmic transformation improved distributional symmetry and reduced the influence of extreme values, supporting its use in regression and ROC analyses. Frailty scores clustered mainly within moderate-to-severe frailty categories, reinforcing that adverse outcomes in this cohort may reflect broader geriatric vulnerability rather than isolated cardiac dysfunction alone.

Secondly, older age was significantly associated with adverse outcomes during univariate analysis, with patients experiencing death or readmission demonstrating substantially higher mean age compared with those without adverse outcomes. This was previously demonstrated by Cendrós et al. [8] in non-Caucasian population studies.

Thirdly, the ROC analysis demonstrated acceptable discriminatory performance of admission NT-proBNP for predicting adverse outcomes, with an AUC of approximately 0.70. The difference in AUC between the two models should be interpreted in the context of the different analytic populations. The initial model was evaluated in 81 patients with complete data, whereas the fully adjusted model was restricted to 53 patients with complete data for all covariates. Therefore, the AUCs were derived from different patient populations and should not be interpreted as a direct measure of the incremental discriminatory value of AF and EF.

The study done by Suzuki and Sugiyama [9] demonstrated the clinical utility of the molar NT-proBNP/BNP ratio as a robust biomarker for predicting heart failure events and assessing complex clinical cohorts, offering superior risk stratification for stable outpatients compared to conventional single-peptide assessments. Beyond mapping the dynamic balance between peptide production and clearance, this composite metric standardizes diagnostic interpretation across diverse patient profiles influenced by age, atrial rhythm, or varying renal function. Furthermore, integrating the NT-proBNP/BNP ratio into longitudinal monitoring provides a more sensitive mechanism to track heart failure progression, evaluate intervention efficacy, and ensure accurate risk stratification when extrinsic factors confound absolute peptide levels. Even though our retrospective cohort study did not assess the effectiveness of the proBNP/BNP molar ratio, the study itself validates the importance of natriuretic peptides as valuable tools for risk stratification when evaluating complex clinical cohorts. The ROC plots visually reinforced this finding, as the curves remained above the diagonal reference line, indicating discrimination better than chance. This suggests that admission NT-proBNP may contribute to identifying higher-risk patients, even though it was not an independent predictor in the adjusted logistic regression model.

Therefore, our findings suggest that NT-proBNP may have greater utility when interpreted alongside other clinical risk factors rather than as a standalone prognostic marker. However, we did not directly compare multivariable prediction models with and without NT-proBNP in this study; this will be investigated in future studies.

The finding that older age was associated with increased risk of death or heart failure admission is consistent with previous heart failure literature demonstrating the prognostic importance of advanced age in acute heart failure populations [8,9]. Older adults with heart failure frequently exhibit reduced physiological reserve, increased multimorbidity burden, impaired functional capacity, and greater vulnerability to clinical deterioration. Within the present cohort, patients with adverse outcomes were approximately six years older on average than patients without adverse events, suggesting that age-related vulnerability may play a substantial role in short-term prognosis. This is again a mirror of the study by Sunaga et al. [10].

Although admission NT-proBNP was not independently significant in the multivariable logistic regression model, higher NT-proBNP levels were consistently observed among patients who experienced adverse outcomes. Patients with death or readmission demonstrated higher mean log-transformed NT-proBNP values than patients without adverse outcomes, supporting the biological plausibility of increased neurohormonal activation in clinically vulnerable patients. This result was directly consistent with the same study results done by de Terwangne et al. [11] on the BELFRAIL cohort. Importantly, in these studies, ROC analysis demonstrated acceptable discriminatory performance of NT-proBNP, suggesting that the biomarker retains clinically useful prognostic information despite the absence of independent statistical significance after adjustment.

This distinction between discriminatory ability and independent multivariable association is clinically important. In frail older adults with acute heart failure, outcomes are unlikely to be driven by a single isolated biomarker. Instead, prognosis probably reflects complex interactions between frailty, age, comorbidity burden, renal function, hemodynamic instability, and reduced physiological reserve. Consequently, NT-proBNP may remain useful as a risk stratification tool even when its prognostic influence becomes attenuated after adjustment for overlapping clinical variables.

The findings relating to frailty are also clinically relevant. Patients experiencing adverse outcomes demonstrated higher CFS scores compared with patients without adverse events, with a trend toward statistical significance. Frailty has increasingly emerged as an important determinant of prognosis in older heart failure populations, particularly among hospitalized patients. Frail individuals frequently exhibit impaired resilience to acute physiological stressors, reduced recovery potential, sarcopenia, nutritional vulnerability, and higher susceptibility to rehospitalization [12]. The directional relationship observed in the present study supports the growing recognition that frailty contributes meaningfully to short-term heart failure outcomes.

Interestingly, EF phenotype was not significantly associated with adverse outcomes. Rates of death or readmission were relatively similar across the HFpEF, HFmrEF, and HFrEF groups. This finding may suggest that in frail older inpatient populations, traditional distinctions between preserved and reduced EF become less prognostically dominant than overall geriatric vulnerability and multimorbidity burden. Previous studies have similarly demonstrated that older adults with HFpEF often experience substantial symptom burden, frailty, and rehospitalization risk despite preserved systolic function. More specifically, Talha et al. [12] showed that vulnerability to acute stressors often eclipses isolated ventricular metrics, which is further confirmed by these study results.

Similarly, AF demonstrated a clinically noticeable but statistically non-significant trend toward increased adverse outcomes. Patients with AF experienced higher proportions of death or readmission compared with those without AF. This observation is biologically plausible given the established relationship between AF, worsening hemodynamic function, elevated natriuretic peptide concentrations, and recurrent hospitalization in heart failure populations. Similar results were observed in the study by Ahmed et al. [13], which provides direct, empirical backing for the results shown in our statistical analysis that confirms the association of AF with a higher risk of hospital readmission in older heart failure patients.

The absence of statistically significant independent predictors within the multivariable regression model should be interpreted cautiously. Several predictors demonstrated clinically coherent directional effects despite non-significant p-values, and CIs were relatively wide. This pattern likely reflects limited statistical power related to the modest sample size and missing data within a heterogeneous retrospective cohort. Consequently, the findings should not necessarily be interpreted as evidence of absent clinical association, but rather as an indication that prognostic relationships within frail older heart failure populations are multifactorial and complex.

The present study has several clinical implications. Admission NT-proBNP may provide useful early risk stratification information in frail older adults hospitalized with acute heart failure, particularly when interpreted alongside measures of frailty and overall clinical vulnerability. The findings additionally support the importance of incorporating geriatric assessment principles into heart failure prognostication, rather than relying exclusively on conventional cardiac biomarkers or EF measurements.

Several limitations should also be acknowledged. The retrospective, single-center design may limit the potential for residual confounding. Important clinical factors that were not consistently available in the medical records, including heart failure therapies and degree of medication optimization, severity of congestion, and functional status, could have influenced short-term outcomes and may not have been fully accounted for in multivariable analysis.

Missing data reduced the sample size available for multivariable modeling. Within the 98-patient cohort that met the inclusion and exclusion criteria, data were missing for 12 patients (12.2%) for NT-proBNP and 33 patients (33.7%) for EF phenotype, while age, sex, CFS, presence of AF, and CKD data were complete. Because the study used retrospectively collected clinical data, the mechanism of missingness could not be formally established. Therefore, missingness cannot be assumed to have been completely at random, particularly where investigations were obtained according to clinical practice. Consequently, informative missingness and associated selection bias cannot be excluded.

Furthermore, the four-month narrow enrolment window (September-December 2024) raises the possibility of seasonal confounding (winter months can have more exacerbations), which is not addressed in this study. We are hoping to assess further audit data on different seasons of the year in the subsequent studies. Missing data reduced the sample size available for multivariable modeling, potentially limiting statistical power and increasing the risk of type II error. In addition, several variables contained incomplete clinical or echocardiographic data because the study relied on routinely collected retrospective hospital records.

A formal a priori sample size calculation was not performed because this was a retrospective observational study based on all eligible patients identified within the predefined study period. Consequently, the available cohort size was determined by the number of eligible cases rather than by a prespecified recruitment target. We acknowledge that the resulting sample size, particularly the reduced number of complete cases available for multivariable analysis and the limited number of outcome events, may have constrained statistical power and increased the risk of imprecise or unstable effect estimates. The number of predictors included in the multivariable model was therefore interpreted in the context of the available outcome events, and the regression findings are considered exploratory and hypothesis-generating.

Consequently, some subgroup and regression analyses were performed using smaller effective sample sizes following complete-case analysis. Multiple imputation was performed as a sensitivity analysis because complete-case analysis reduced the available sample from 98 to 53 patients. Missing data were present for log-transformed NT-proBNP (12/98, 12.2%) and EF phenotype (33/98, 33.7%), while outcome status, age, CKD, CFS, and AF were complete. Multiple imputation was therefore used to assess the robustness of the multivariable regression findings to missing predictor data and to reduce potential bias associated with exclusion of patients with incomplete covariate information.

In addition, survival analysis could not be performed because time-to-event data were unavailable. As complete-case analysis was performed, missing data may have introduced selection bias if patients with complete investigations or documentation differed systematically from those with incomplete data. We did not perform a separate competing-risk analysis because of the potential for model instability. The composite outcome of death or heart failure readmission should be interpreted with caution, given the competing-risk relationship between its components, as death precludes subsequent readmission and the two outcomes may have distinct clinical determinants. Given the relatively small number of events and the exploratory nature of this analysis, we did not analyze this, as it would make the model more complex. However, we suggest that future studies with larger sample sizes should evaluate death and heart failure readmission separately using appropriate competing-risk methods. Finally, unmeasured clinical factors including treatment variation, functional decline, nutritional status, and social support may also have influenced short-term outcomes.

Despite these limitations, the study possesses several important strengths, including its focus on a frail older inpatient population, the incorporation of frailty assessment alongside biomarker analysis, and the use of multivariable modeling and ROC analysis to evaluate prognostic performance. The findings contribute to the growing literature emphasizing the complexity of prognostication in older adults with acute heart failure.

## Conclusions

This retrospective cohort study of frail older adults hospitalized with acute heart failure reveals that admission NT-proBNP demonstrated modest discriminatory ability for predicting short-term death or heart failure readmission, with an AUC of 0.703. Although no individual variable, including NT-proBNP, age, frailty score, CKD, AF, or EF phenotype, independently predicted adverse outcomes following multivariable adjustment, the analyses identified clinically coherent directional associations across multiple prognostic indicators. Specifically, older age, greater frailty burden, and higher NT-proBNP levels consistently demonstrated meaningful associations with poor prognosis despite not achieving independent statistical significance. This distinction between discriminatory ability and independent multivariable association carries important clinical implications, suggesting that NT-proBNP may retain clinically useful prognostic information when interpreted alongside measures of frailty and overall geriatric vulnerability.

The findings support the view that prognostication in frail adults with heart failure cannot be driven by isolated cardiac biomarkers or EF phenotypes alone. Rather, short-term outcomes are likely determined by multifactorial clinical vulnerability encompassing age-related physiological decline, overall comorbidity burden, neurohormonal activation, renal dysfunction, and reduced physiological reserve. These results emphasize the importance of incorporating comprehensive geriatric assessment principles into heart failure prognostication strategies, rather than relying exclusively on conventional cardiac metrics. Future research should prioritize prospective validation in larger, more diverse populations with complete clinical data capture, and investigate whether integrated risk stratification incorporating both biomarkers and frailty assessments may improve prognostic accuracy and clinical decision-making in this vulnerable patient population.
